# The role of ^18^F-FDG uptake features in the differential diagnosis of solitary pulmonary lesions with PET/CT

**DOI:** 10.1186/s12957-015-0679-2

**Published:** 2015-09-15

**Authors:** Ming Zhao, Baolin Chang, Zhihua Wei, Hongtao Yu, Rongrong Tian, Ling Yuan, Hongxing Jin

**Affiliations:** Department of PET/CT Center, The Tumor Hospital of Shanxi Province, No.3, Zhigongxin Street, 030013 Taiyuan, China; Department of Thoracic Surgery, The Tumor Hospital of Shanxi Province, No.3, Zhigongxin Street, 030013 Taiyuan, China; Department of Medical Imaging, Shanxi Medical University, Taiyuan, China

**Keywords:** Solitary pulmonary lesions, ^18^F-FDG, PET/CT

## Abstract

**Background:**

The aim of this study is to evaluate the value of ^18^F-FDG uptake features in the diagnosis of solitary pulmonary lesions.

**Methods:**

One hundred thirty-nine patients with solitary pulmonary lesions were divided into full uptake, circular uptake, multi-focus uptake, mild uptake, and no-uptake groups according to the uptake features of ^18^F-FDG in solitary pulmonary lesions. The incidence of benign and malignant lesions and the false-positive and false-negative rates in each group were analyzed. The sensitivity, specificity, accuracy, positive predictive value (PPV), and negative predictive value (NPV) of the method using ^18^F-FDG uptake features combined with maximum standard uptake value (SUVmax) (SUV method) in the differential diagnosis of solitary pulmonary lesions were evaluated.

**Results:**

There were 89 malignant and 50 benign lesions. (1) The malignant incidence of the full uptake group was 84.0 % (63/75), and there were significant differences when compared with the other groups except the circular uptake group (16/23) (all *P* = 0.0001). The benign incidence of the multi-focus and no-uptake groups was 83.3 % (10/12) and 82.4 % (14/17), respectively, and there were significant differences when compared with the full uptake and the circular uptake groups, respectively (all *P* < 0.05). The benign incidence of the mild uptake group was 58.3 % (7/12), and there were no significant differences when compared with the others except the full uptake group (all *P* > 0.05). No statistical significance was found between either two of the no-uptake, mild uptake, and multi-focus uptake groups (all *P* > 0.05). (2) In cases with SUVmax ≥2.5, the false-positive rate in the multi-focus uptake group was 83.3 % (10/12), which was significantly higher than in the full uptake (12/75) or circular uptake group (7/23) (all *P* < 0.05). In cases with SUVmax <2.5, the false-negative rates in the mild and no-uptake groups were 41.7 and 17.6 % (*P* = 0.218). (3) The sensitivity, specificity, accuracy, PPV, and NPV of the method using ^18^F-FDG uptake features combined with SUVmax and the single SUV method were 88.7 %/91.0 %, 62.0 %/42.0 %, 79.1 %/73.4 %, 80.6 %/73.6 %, and 75.6 %/72.4 %, respectively.

**Conclusions:**

The method using uptake features of ^18^F-FDG combined with SUVmax can improve the diagnostic specificity and accuracy of solitary pulmonary lesions. The multi-focus uptake feature maybe a benign sign, which still needs more researches to confirm.

## Background

The differential diagnosis of a solitary pulmonary lesion includes neoplasm, inflammatory pseudotumor, chronic inflammatory granulomatous disease, tuberculosis, cartilaginous hamartoma, etc. [1–3]. It is well known that the value of ^18^F-FDG PET/CT imaging in the diagnosis of solitary pulmonary lesions had been affirmed for years and a maximum standard uptake value (SUVmax) of ≥2.5 was used as a cutoff point for detecting malignancy [4] (SUV method). However, we found in our daily work that ^18^F-FDG PET/CT can not only provide the standard uptake values (SUV) but also provide the features of ^18^F-FDG accumulation. The features of ^18^F-FDG accumulation, to a certain extent, may be related to the distribution of tumor cells or other nonspecific inflammatory cells in the lesions. However, to the best of our knowledge, there has been no report in the literature regarding the uptake features of ^18^F-FDG. We therefore analyzed the ^18^F-FDG PET/CT imaging of 139 patients with solitary pulmonary lesions in order to evaluate the diagnostic value of ^18^F-FDG uptake classification features.

## Methods

### Patients

We set up a classification according to the ^18^F-FDG uptake features in the solitary pulmonary lesions in September 2011. We consecutively enrolled 121 patients identified with solitary pulmonary lesions (≥8 mm) using ^18^F-FDG PET/CT scan from September 2011 to October 2014. In addition, another 18 patients with identified solitary pulmonary lesions (≥8 mm) during the period of September 2010 to August 2011 were retrospectively analyzed by this classification. Therefore, in total, this study included a cohort of 139 patients.

All the patients had radiologic evidence of single pulmonary nodule or mass with no definite diagnosis or specific treatment before PET/CT scan. None of the patients had mediastinal adenopathy, atelectasis, or other abnormal lesions within the lung. All patients had pathological diagnosis via surgical processes (operation or biopsy) or clinical findings via imaging during a follow-up period of at least 2 years.

### Ethics, consent, and permissions

This study was approved by the hospital medical ethic committee. Consents to participate the study from the participants (or legal parent or guardian for children) were obtained.

### Consent to publish

We had obtained the consents to publish from the participant (or legal parent or guardian for children) to report individual patient’s data in any form (including images, videos, voice recordings, etc.).

### Imaging protocols

#### Equipment and reagents

The PET/CT imaging was carried out on a Discovery STE 16-slice PET/CT scanner (GE Healthcare, USA) at the Tumor Hospital of Shanxi Province. ^18^F (fluorine-18) was produced by Minitrace cyclotron (GE, USA), and FDG (deoxidized fluoride glucose) kit was purchased from Jiangsu HuaYi Technology LTD (GE, USA). ^18^F-FDG (2-^18^F-fluoro-2-deoxy-d-glucose) was synthesized using a TRACERLAB FXN multifunctional synthesizer. The radiochemical purity was at least 90 %.

#### PET/CT scanning

All patients fasted for at least 4 h before ^18^F-FDG administration. When blood glucose was <11 mmol/L, 5–6 MBq of ^18^F-FDG per kilogram of body weight was intravenously administered. The images were acquired 50 min after injection. Low-dose scan (tube voltage 120 kV; effective tube current 10 mA) was performed for attenuation correction. A low-dose CT scan (tube voltage 120 kV; effective tube current 180 mA) was obtained from the skull to the femoral upper middle section for identifying the lesion’s precise anatomical location when the patient was in supine position and breathing quietly. The CT data were reconstructed by filtered back projection (FBP) into 512 × 512 pixel images with a slice thickness of 3.75 mm to match the PET. The PET data were acquired on the GE Discovery STE in 3-dimensional mode, with 6 or 7 bed positions and 3 min per bed position, and the scan scope was consistent with CT. The PET was reconstructed by ordered subsets expectation maximization (OSEM). The CT, PET, and PET/CT fusion images were reviewed in all standard planes with maximum-intensity whole-body projection images on an AW workstation (GE Healthcare).

### Imaging analysis

#### The calculation of the SUV

The trans-axial image with the highest ^18^F-FDG uptake in the solitary pulmonary lesions was selected. A circular region of interest (ROI) of the whole lesion was sketched. The SUVmax of the ROI was calculated. The SUV was calculated as the regional radioactivity concentration divided by the injected amount of radioactivity normalized by body weight.

### Imaging reading

^18^F-FDG PET/CT images were evaluated retrospectively and read separately by two experienced nuclear medical physicians who were unaware of the patient’s name, follow-up, and pathological findings. If they had disagreements, images would be read by another three experienced nuclear medical physicians. Consensus was reached among at least three of five readers.

### Experimental groups and the analysis of the false-negative and false-positive cases

**Fig. 1:**
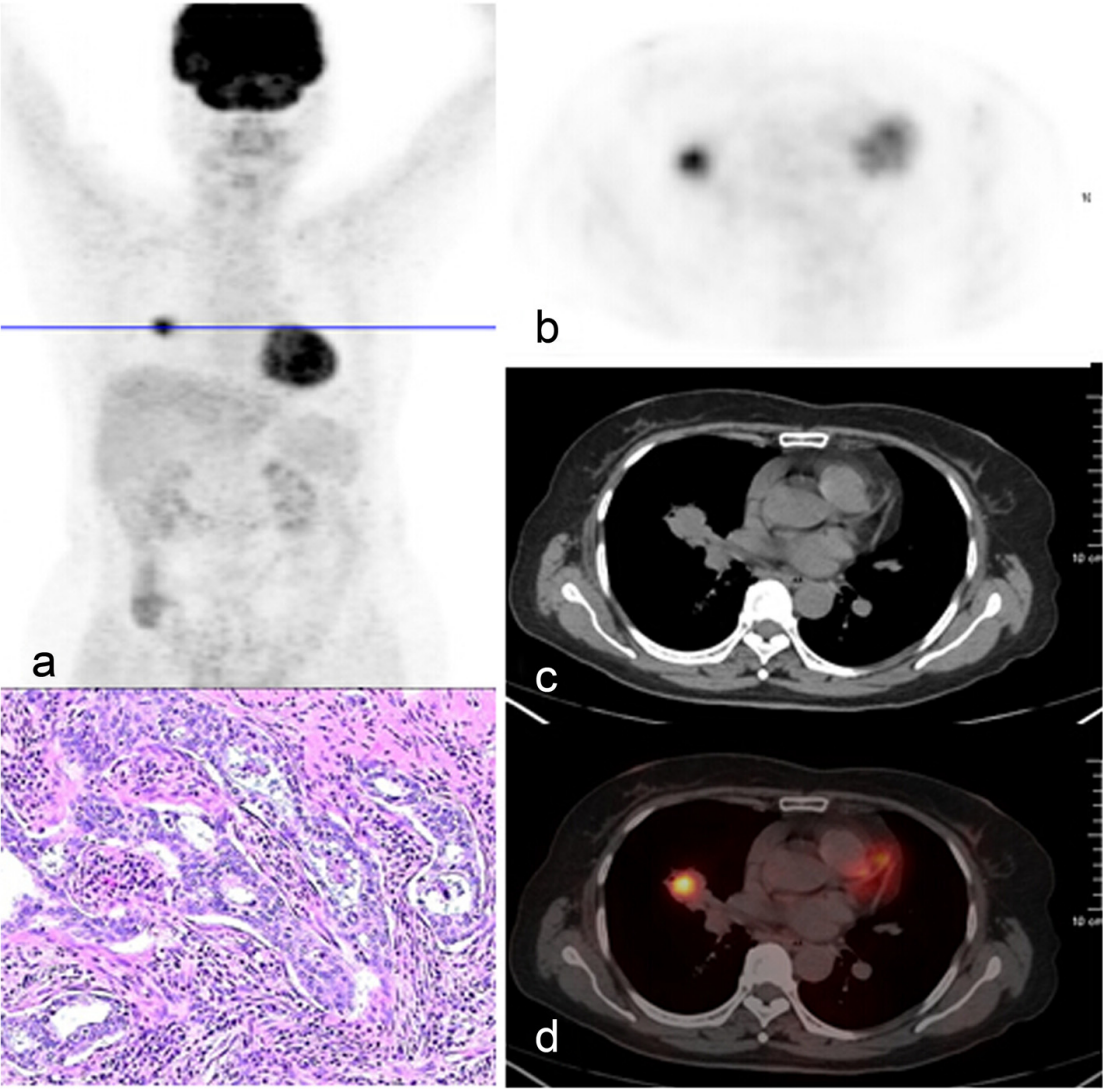
Full ^18^F-FDG uptake nodule in a 68-year-old female. The whole-body PET (**a**) and axial PET (**b**), CT (**c**), and fused PET/CT images (**d**) show FDG accumulation in the nodule of the middle lobe of the right lung. The pathologic result was adenosquamous carcinoma

**Fig. 2:**
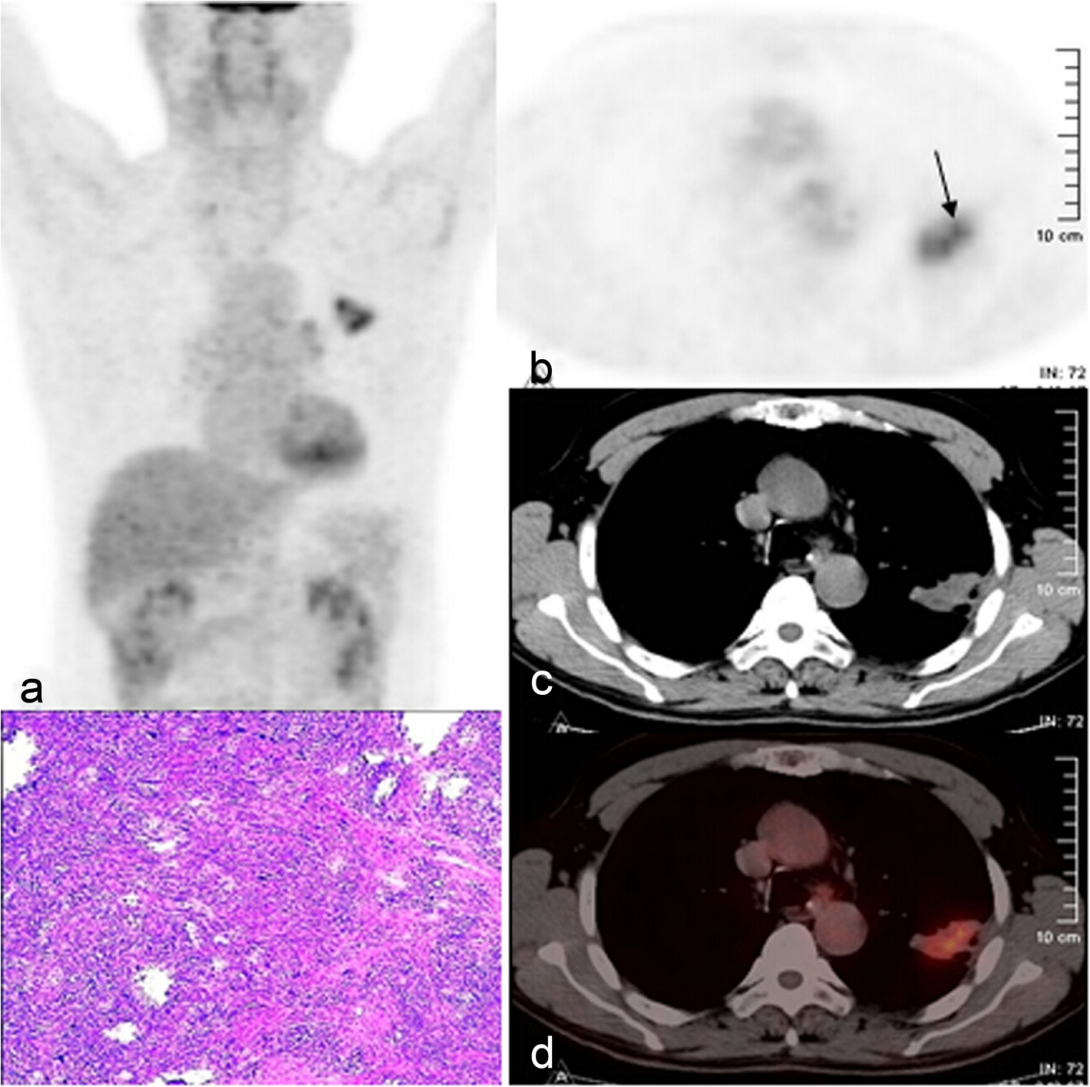
Multi-focus ^18^F-FDG uptake mass in a 65-year-old male. The whole-body PET (**a**) and axial PET (**b**), CT (**c**), and fused PET/CT images (**d**) show multi-focus FDG accumulation in the mass of the left upper lung lobe (*black arrow*). The pathologic result was inflammatory pseudotumor

According to the uptake features of the lesions, patients were divided into five groups: (1) the no-uptake group, which was defined as the FDG uptake of the lesion no higher than the background of the lung (*n* = 17); (2) mild uptake group: the FDG uptake of the lesion was higher than the background, but SUVmax was lower than 2.5 (*n* = 12); (3) full uptake group (Fig. 1): more than two consecutive PET trans-axial images of the lesion were matched with CT images completely, and the SUVmax was equal to or higher than 2.5 (*n* = 75); (4) circular uptake group: FDG uptake of the center was lower than the periphery of the lesion, and the SUVmax was equal to or higher than 2.5 (*n* = 23); and (5) multi-focus uptake group (Fig. 2): some of the patients identified with solitary pulmonary lesions on CT scans were found to have uneven high FDG uptake with two or more increased uptake focuses in it on PET section at the same corresponding level and 3D PET images, and have a SUVmax ≥2.5 (*n* = 12). The incidence of benign and malignant lesions and the false-positive and false-negative rates in each group were analyzed.

### The diagnosis of solitary pulmonary lesions by the ^18^F-FDG uptake feature classification

The sensitivity, specificity, accuracy, and false-positive and false-negative rates were analyzed using ^18^F-FDG uptake feature classification, and the results were compared with those from using the SUV method.

### Statistical analysis

The SPSS 17.0 statistical analysis software (SPSS Inc., Chicago, IL, USA) was used, and the data were expressed as mean ± standard deviation. The *χ*^2^ test and Fisher’s exact test were used to compare the incidences of benign or malignant lesions in different groups. ROC curve was used to compare the diagnosis accuracy of the two methods. *P* < 0.05 was considered as statistically significant.

## Results

### Clinical results

Among the 139 cases of solitary pulmonary lesions enrolled in this study, 87 were males and 52 were females. Patients’ ages ranged from 25 to 85 years, with a mean age of 62.61 ± 10.17 years. One hundred eighteen patients had surgery or biopsy, and 21 cases had CT morphologic examination during follow-up at 2 years after PET/CT scan. Among them, 89 lesions were malignant and 29 were benign tumors confirmed by evaluation of resection specimens or biopsy, and the other 21 cases were confirmed benign by morphologic stability on CT. Eighty lesions were in the right lung, and 59 were in the left lung. The maximum diameters of the lesions ranged from 0.8 to 12.9 cm (mean 4.43 ± 2.37) (Table 1).

**Table 1:** The range of maximum diameter of lesions in each group

	Total	FUG	CUG	MFUG	MUG	NUG
Range (cm)	0.8–12.9	0.9–7.2	2.5–12.9	2.2–6.9	1.3–6.0	0.8–6.4
Mean ± SD	4.43 ± 2.37	3.38 ± 1.45	6.66 ± 2.66	4.45 ± 1.46	3.65 ± 1.68	3.67 ± 1.85

*FUG* full up take group, *CUG* circular uptake group, *MFUG* multi-focus uptake group, *MUG* mild uptake group, *NUG* no-uptake group

### The pathological findings and follow-up results

The 89 malignant cases included 59 cases of adenocarcinoma, 16 of squamous cell carcinoma, 2 of adenosquamous carcinoma, 7 of small cell lung cancer, 4 of neuroendocrine carcinoma, and 1 of primitive neuroectodermal tumor (PNET). The 29 benign cases included 10 cases of inflammatory pseudotumor, 7 of tuberculosis, 4 of lung abscess, 2 of isolated fibrous tumor, 1 of sclerosing hemangioma, 1 of neurilemmoma, and 4 of cartilaginous hamartoma. The rest were confirmed benign during a follow-up period of at least 2 years with no change in lesion size or shape on CT (Table 2).

**Table 2:** The pathologic results and follow-up results of solitary pulmonary lesions in each group

Groups	Total	FUG	CUG	MFUG	MUG	NUG
Malignant	89	63	16	2	5	3
Adenocarcinoma	59	40	11	2	5	1
Squamous cell carcinoma	16	12	4			
Adenosquamous carcinoma	2	1	1			
Small cell lung cancer	7	7				
Neuroendocrine carcinoma	4	3				1
Primitive neuroectodermal tumor	1					1
Benign	50	12	7	10	7	14
Inflammatory pseudotumor	10	5	1	3	1	
Tuberculosis	7	2	1	2	1	1
Lung abscess	4		4			
Cartilaginous hamartoma	4			1	1	2
Isolated fibrous tumor	2					2
Sclerosing hemangioma	1				1	
Neurilemmoma	1	1				
Benign in the follow-up period	21	4	1	4	3	9

*FUG* full up take group, *CUG* circular uptake group, *MFUG* multi-focus uptake group, *MUG* mild uptake group, *NUG* no-uptake group

### Analysis of the incidence of benign or malignant lesions and the false-positive and false-negative rates in each group

These cases were divided into no-uptake, mild uptake, full uptake, circular uptake, and multi-focus uptake groups according to the ^18^F-FDG uptake features of the lesions. The SUVmaxs of the full uptake, circular uptake, and multi-focus uptake groups were higher than or equal to 2.5, while the SUVmaxs of the no-uptake and mild uptake groups were lower than 2.5. The incidence of malignant lesions was arranged as follows in descending order: full uptake (63/75) > circular uptake (16/23) > mild uptake (5/12) > no-uptake (3/17) > multi-focus uptake (2/12) groups. The malignant incidence of the full uptake group was 84.0 %, and there were significant differences when compared with the mild uptake group (*χ*^2^ = 8.522, *P* = 0.001), no-uptake group (*χ*^2^ = 26.912, *P* = 0.0001), and the multi-focus uptake group (*χ*^2^ = 21.389, *P* = 0.0001), respectively; there was no significant difference when compared with the circular uptake group (*χ*^2^ = 1.514, *P* = 0.219). The malignant incidence of the full uptake group combining with the circular uptake group was 80.6 % (79/98), and there were significant differences when compared with the mild uptake group (*χ*^2^ = 6.956, *P* = 0.008), no-uptake group (*χ*^2^ = 25.077, *P* = 0.0001), and the multi-focus uptake group (*χ*^2^ = 19.345, *P* = 0.0001), respectively.

The incidence of benign lesions was arranged as follows in descending order: multi-focus uptake (10/12) > no-uptake (14/17) > mild uptake (7/12) > circular uptake (7/23) > full uptake (12/75) groups. The benign incidence of the multi-focus uptake group was 83.3 % (10/12), and no statistical significances were found between either two of the no-uptake, mild uptake, and multi-focus uptake groups (all *P* > 0.05). The rate of benign incidences in the no-uptake group combining with the multi-focus uptake group was 82.8 % (24/29), which were significantly higher than the full uptake group and circular uptake group (*χ*^2^ = 41.181, *P* = 0.0001; *χ*^2^ = 14.586, *P* = 0.0001), while there was no significant difference when compared with the mild uptake group (*χ*^2^ = 1.581, *P* = 0.209).

In the SUVmax ≥2.5 groups, the false-positive rates in the full uptake group, circular uptake group, and multi-focus uptake group were 16.0 % (12/75), 30.4 % (7/23), and 83.3 % (10/12), respectively, when the SUV method was used. The multi-focus uptake group had the highest false-positive rate, which was significantly different from those in the full uptake group or the circular uptake group (*χ*^2^ = 21.389, *P* = 0.0001; *P* = 0.005. respectively). In the cases of SUVmax <2.5, the false-negative rates of mild uptake group and no-uptake group were 41.7 % (5/12) and 17.6 % (3/17), respectively. The difference was not statistically significant (*P* = 0.218) (Table 3).

**Table 3:** The diagnostic results of the benign and malignant lesions of each group

Groups	Total	Malignant		Benign		False-positive rate	Accuracy
	*n*	*n*	%	*n*	%	%	%
SUVmax ≥2.5	110	81	73.6	29	26.4	26.4	73.6
FUG	75	63	84.0	12	16.0	16.0*	84.0
CUG	23	16	69.6	7	30.4	30.4^#^	69.6
MFOG	12	2	16.7	10	83.3	83.3*^#^	16.7
Groups	Total	Malignant		Benign		False-negative rate	Accuracy
	*n*	*n*	%	*n*	%	%	%
SUVave <2.5	29	8	27.4	21	72.6	27.4	72.6
MUG	12	5	41.7	7	58.3	41.7	58.3
NUG	17	3	17.6	14	82.4	17.6	82.4

* P < 0.05, ^#^ P < 0.05, ^#^* The false-positive rate in MFOG was significantly different from that in FUG or CUG, but there was no significant difference between CUG and FUG

### Comparison of the diagnostic results by using the uptake feature classification and the single SUV method

Based on the results above, the full and circular uptake groups were defined as the determined malignant group and the no-uptake, mild uptake, and multi-focus uptake groups were defined as the determined benign group in this study. There were 79 malignant cases and 19 benign cases in the determined malignant group, and the incidence of malignant lesions was 80.6 % (79/98). There were 10 malignant cases and 31 benign cases in the determined benign group, and the incidence of malignant lesions was 24.4 % (10/41). The difference between these two groups was statistically significant (*χ*^2^ = 39.671, *P* = 0.0001).

**Fig. 3:**
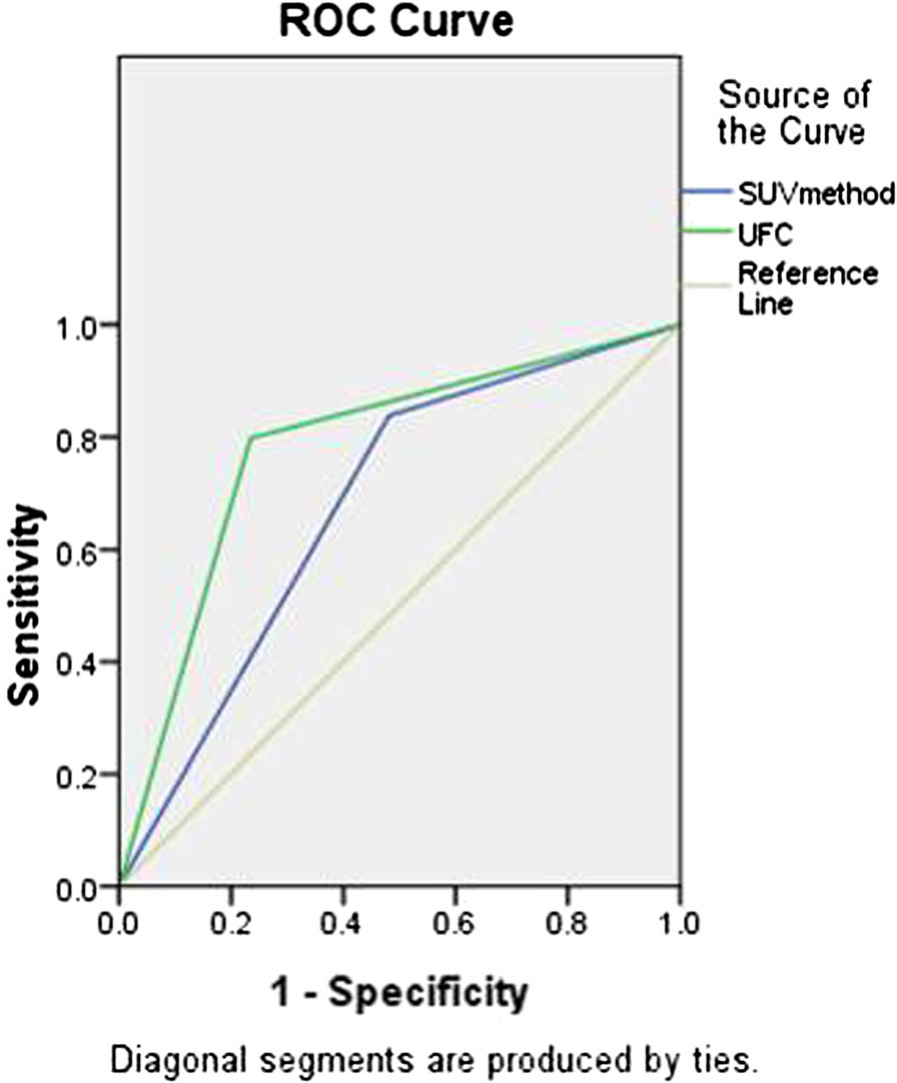
ROC curve. The *green curve* represents the uptake feature classification, and the *blue curve* represents the SUV method of PET/CT. The two curves were based on the nature of the lesions with benign or malignant interpretation. AUC was 0.678 for SUV method and 0.782 for the uptake feature classification

The diagnostic sensitivity, specificity, accuracy, positive predictive value (PPV), and negative predictive value (NPV) for the uptake feature classification combining with the SUV method were 88.8 % (79/89), 62.0 % (31/50), 79.1 % (110/139), 80.6 % (79/98), and 75.6 % (31/41), respectively (Table 4, Fig. 3).

**Table 4:** The results of different diagnostic methods of PET/CT in characterization of 139 solitary pulmonary lesions

Methods	TP	TN	FP	FN	Accuracy	Sensitivity	Specificity	PPV	NPV
	*n*	*n*	*n*	*n*	%	%	%	%	%
SUV	81	21	29	8	73.4	91.0	42.0	73.6	72.4
UFC	79	31	19	10	79.1	88.7	62.0	80.6	75.6

*TP* true-positive, *TN* true-negative, *FP* false-positive, *FN* false-negative, *PPV* positive predictive value, *NPV* negative predictive value, *UFC* uptake feature classification

Using the single SUV method, there were 81 malignant and 29 benign cases in the group with SUVmax ≥2.5, and the incidence of malignant lesions was 73.6 % (81/110). There were 8 malignant and 21 benign cases in the group with SUVmax <2.5, and the incidence of malignant lesions was 27.4 % (8/29). The difference between these two groups was statistically significant (*χ*^2^ = 21.130, *P* = 0.0001).

The diagnostic sensitivity, specificity, accuracy, PPV, and NPV for the SUV method were 91.0 % (81/89), 42.0 % (21/50), 73.4 % (102/139), 73.6 % (81/110), and 72.4 % (21/29), respectively.

## Discussion

Solitary pulmonary nodule (SPN) is defined as a single spherical or oval lesion completely surrounded by the lung, 3 cm or less in diameter, without associated atelectasis, lymph node enlargement, or other abnormal lesions within the lung [5]. Lesions measuring greater than 3 cm are classified as masses [6]. The solitary lesions in this study ranged from 0.8 to 12.9 cm and were collectively referred to as solitary pulmonary lesions.

The estimated prevalence of solitary pulmonary nodules in the literature ranges from 8 to 51 % [7]. Previous studies found that 25–39 % of malignant nodules were inaccurately classified as benign by CT assessment [8], and over 50 % of the radiographically indeterminate nodules resected at thoracoscopy were benign [9]. It is thus critical to diagnose these lesions accurately. A transthoracic needle aspiration biopsy has a higher diagnostic yield, but can be complicated by a pneumothorax, requiring drainage in 5–10 % of procedures [10]. Moreover, the technique is still hampered by the possibility of a false-negative test result, which carries the risk of an unacceptable expectation in patients with early-stage lung cancer [11, 12].

The value of ^18^F-FDG PET imaging in the diagnosis of solitary pulmonary nodules had been confirmed. ^18^F-FDG PET scan can distinguish the benign and malignant lesions depending on the uptake value of FDG. In a meta-analysis, Gould et al. found a sensitivity of 94 % and a specificity of 86 % in characterization of solitary pulmonary nodules and mass lesions (<4 cm in size) using SUVave of ≥2.5 as a cutoff point for detecting malignancy [13]. A review article by Vansteenkiste and Stroobants indicates that for the SUV method, the sensitivity, specificity, and accuracy were 96 % (range 83–100), 79 % (range 52–100), and 91 % (range 86–100), respectively [9]. The variations are likely due to differences in the prevalence of malignancy in the study populations. In this study, the sensitivity, specificity, and accuracy were 91.0 % (81/89), 42.0 % (21/50), and 73.4 % (102/139) for ^18^F-FDG PET/CT using the SUV method. The sensitivity was similar to those reported earlier [9], while the specificity and accuracy were lower. It is plausible to postulate that the main reason for this disparity is the relatively high prevalence of inflammation and TB in our study population, which resulted in a higher false-positive rate and a lower specificity. Our data are consistent with the report by Sebro and coworkers [14].

Nonspecific ^18^F-FDG uptake can be seen in bacterial pneumonia, pyogenic abscess, chronic granulomatous disease [15–17], etc. In these lesions, the ^18^F-FDG uptake has been attributed to an increase in granulocyte and/or macrophage activity [18]. When the SUV diagnostic method was used, there were 29 false-positive cases, including 5 cases of tuberculosis, 9 of inflammatory pseudotumor, 4 of lung abscess, 1 of cartilaginous hamartoma, 1 of neurilemmoma, and the other 9 cases were confirmed to be benign during the follow-up period. The false-positive rate in the multi-focus uptake group was 83.3 %, which was significantly higher than the other groups (Table 2). The pathology and follow-up results in this group indicated 3 cases of inflammatory pseudotumor, 2 of tuberculosis, 1 of cartilaginous hamartoma, and the other 4 cases were still being followed up for inflammation. Inflammatory pseudotumor is mixed by inflammatory cells (including plasma cells, lymphocytes, histiocytes, foam cells, multinucleated giant cells, etc) and spindle mesenchymal cells (including myofibroblast, fibroblast, collagen fibers) in different ratios. Tuberculosis is composed of epithelioid cells and/or Langhans cells, caseous necrosis, and varying amounts of lymphocytes. And, the distribution and the growth of pathogenic bacteria swallowed by the alveolar macrophages or other inflammatory cells are always multifocal, which may be related to the characteristic of the multi-focus uptake. The minimum diameter with the multi-focus uptake observed in this study was 2.2 cm.

It is noteworthy that there were still some malignant lesions in the SUVmax <2.5 cases, most of which were well-differentiated adenocarcinomas and alveolar carcinoma, and part of which were neuroendocrine tumors [19–21], which always made misdiagnosis [22]. The false-negative rate in the mild uptake group was 41.7 % (5/12), while the false-negative rate in the no-uptake group was 17.6 % (3/17). Although there was no statistical significance, the false-negative cases were mainly encountered in the mild uptake group when pulmonary lesions were diagnosed by the single SUV method. The postoperative pathological results of the 5 false-negative cases in mild uptake group were all adenocarcinomas (2 cases of early adenocarcinoma and 3 cases of adenocarcinoma of class II).

Kim et al. analyzed 42 cases of 0.7–3 cm SPNs by using the SUV method combining with the CT scores. They found that PET/CT significantly reduced the false-positive rate and improved the specificity compared with CT alone, and PET/CT significantly reduced the false-negative rate while the number of false-positive cases had no change when compared with PET alone. The sensitivity and accuracy of PET/CT in the diagnosis of malignant SPNs (97 %/93 %, respectively) were significantly superior to PET (69 %/74 %), although the specificity did not improve obviously (both 85 %) [23].

When we used the SUV method to diagnose the 139 solitary pulmonary lesions, the sensitivity, specificity, accuracy, PPV, and NPV of PET/CT were 91.0 % (81/89), 42.0 % (21/50), 73.4 % (102/139), 73.6 % (81/110), and 72.4 % (21/29), respectively. However, when the multi-focus ^18^F-FDG uptake group was defined as the benign group although the SUVmax was higher than 2.5, the sensitivity, specificity, accuracy, PPV, and NPV of 139 solitary pulmonary lesions were 88.8 % (79/89), 62.0 % (31/50), 79.1 % (110/139), 80.6 % (79/98), and 75.6 % (31/41), respectively. The specificity, accuracy, PPV, and NPV were all improved compared with the single SUV method, while the sensitivity reduced slightly (Fig. 3).

## Conclusions

Our study showed that the uptake feature classification improved the diagnostic specificity and declined the false-positive rate of ^18^F-FDG PET/CT for solitary pulmonary lesions compared with the single SUV method. And, it still needs larger samples and multicenter researches to confirm whether the multi-focus ^18^F-FDG uptake feature is an important sign of chronic inflammatory pseudotumor or tuberculosis in the differential diagnosis of solitary pulmonary lesions.

## Abbreviations

FBP: filtered back projection
OSEM: ordered subsets expectation maximization
PNET: primitive neuroectodermal tumor
ROI: region of interest
SPN: solitary pulmonary nodule
SUV: standard uptake value
SUVmax: maximum standard uptake value
